# Correction to: Chromosome-Level Assemblies of the *Pieris mannii* Butterfly Genome Suggest Z-Origin and Rapid Evolution of the W Chromosome

**DOI:** 10.1093/gbe/evad224

**Published:** 2023-12-14

This is a correction to: Daniel Berner, Simona Ruffener, Lucas A Blattner, Chromosome-Level Assemblies of the *Pieris mannii* Butterfly Genome Suggest Z-Origin and Rapid Evolution of the W Chromosome, *Genome Biology and Evolution*, Volume 15, Issue 6, June 2023, evad111, https://doi.org/10.1093/gbe/evad111

In the originally published version of this manuscript, the funding statement for this article was missing. Hence the sentence ‘Financial support was provided by the Swiss National Science Foundation (grant 310030_200374 to D.B.).’ has been added to the Acknowledgments section.

This error has been corrected online.

